# Exploration Behaviour Is Not Associated with Chick Provisioning in Great Tits

**DOI:** 10.1371/journal.pone.0026383

**Published:** 2011-10-20

**Authors:** Samantha C. Patrick, Lucy E. Browning

**Affiliations:** 1 Edward Grey Institute, University of Oxford, Oxford, United Kingdom; 2 Marine Biology and Ecology Research Centre, University of Plymouth, Plymouth, United Kingdom; 3 Arid Zone Research Station, University of New South Wales, Sydney, Australia; University of Jyväskylä, Finland

## Abstract

In biparental systems, members of the same pair can vary substantially in the amount of parental care they provide to offspring. The extent of this asymmetry should depend on the relative costs and benefits of care. Individual variation in personality is likely to influence this trade-off, and hence is a promising candidate to explain differences in care. In addition, plasticity in parental care may also be associated with personality differences. Using exploration behaviour (EB) as a measure of personality, we investigated these possibilities using both natural and experimental data from a wild population of great tits (*Parus major*). Contrary to predictions, we found no association between EB and natural variation in provisioning behaviour. Nor was EB linked to responsiveness to experimentally increased brood demand. These results are initially surprising given substantial data from other studies suggesting personality should influence investment in parental care. However, they are consistent with a recent study showing selection on EB is weak and highly context-specific in the focal population. This emphasises the difficulty faced by personality studies attempting to make predictions based on previous work, given that personalities often vary among populations of the same species.

## Introduction

The optimal level of parental care should reflect a trade-off between the benefits of maximizing the survival of the current brood and the costs that this investment imposes on future reproductive attempts [1]. Substantial variation exists among species in the extent and form of parental care, much of which can be attributed to variation in life history strategies that determines the nature of this trade-off [2], [3]. In birds, most species are socially monogamous with biparental care [2], [4], [5], a reproductive strategy that is relatively rare in other taxa [3]. Where both parents provide care for their offspring, optimal investment strategies become more complex as parents must incorporate partner contributions. How the burden of care is shared between parents has been the focus of much attention in an effort to understand how sexual conflict over care shapes the provisioning rules used to adjust care facultatively [6], [7], [8], [9]. Indeed, much of the individual-level variation in parental care that exists, arises due to differential investment between the sexes [1], [2], [3]. However, the variation that exists once sex differences have been accounted for remains largely unexplained.

A promising candidate for explaining variation in parental care is the variation in personality that exists among individuals of the same species. Individuals from a wide range of taxa have been found to vary consistently in suites of correlated behavioural traits [10], [11], [12], [13], [14]. When behaviours are found to correlate across individuals, they are described as a ‘behavioural syndrome’ [14]. Over the past decade one of the most comprehensive studies on personality has been carried out on the great tit (*Parus major*) [15]. In this species, how an individual explores a novel environment (exploration behaviour: EB) has been used extensively as a index of personality [16] because it covaries with other ecologically important behaviours [17], [18], [19], [20]. Individuals that explore quickly and superficially are bolder, more aggressive and more likely to take risks that conspecifics that explore slowly but thoroughly [15]. Given that the relative differences in behaviour among individuals remain stable over time and in different contexts, they may alter the costs and benefits of caring for young, yielding personality-specific reproductive strategies [21].

In great tits, there is growing evidence that EB is likely to influence parental care. Fast-exploring birds defend their nest more aggressively than slow explorers [18]. However slow-exploring individuals are better able to exploit new food sources [16], [22], which may enable them to provision young when conditions are harsh. In addition, EB has also been found to correlate with fledging success [23] and offspring recruitment [24], [25]. While the mechanism responsible for these associations remains to be elucidated, a likely explanation is that they are mediated through parental care. It has also been shown that variation in EB is associated with differences in behavioural plasticity [16], [20], [26], whereby slower exploring birds are better able to adjust their behaviour in response to changes in their environment. This may have important consequences for facultative responses in parental care and the interaction between the EB of parents is likely to have implications for how the pair work together to care for young.

In this study, we investigate the association between personality and parental care in a wild population of great tits. We use EB as a measure of personality and quantify parental care by recording provisioning rates of young in the nest. Our aims are threefold. First, we investigate the relationship between EB and individual provisioning rates. Second, we investigate the relationship between EB and the relative contribution of individuals within each pair and third, we investigate the relationship between EB and plasticity in provisioning by experimentally manipulating brood demand. We predict that bolder birds should be able to provision at a higher rate since they may dominate in competitive encounters over food. However, shyer birds should demonstrate greater plasticity in provisioning rate since they are more sensitive to changes in their environment.

## Results

Individual provisioning rates ranged from 1–50 visits per hour (mean ± sd = 14.25±0.12). Provisioning rates were not correlated with EB (EB: *F*_1, 2389_ = 0.31; *p* = 0. 58), nor did this relationship depend on sex (EB×sex: *F*_1, 2389_ = 0.47; *p* = 0.50), year (EB×year: *F*_2, 2387_ = 1.92; *p* = 0.15), or all three (EB×sex×year: *F*_2, 2387_ = 0.31; *p* = 0.73). There was no interaction between EB and hour (EB×hour: F_1, 2388_ = 0.39; p = 0.54). Males and females did not differ in their number of feeds per hour (sex: *F*_1, 2914_ = 0.59; *p* = 0.44), although both parents fed larger broods at a higher rate (brood size: *F*_1, 2914_ = 24.91; *p*<0.0001). There were age differences in provisioning rate, with first year birds feeding at higher rate than adults (age: *F*_1,2914_ = 16.83; *p*<0.0001) but there was no interaction between EB and age (EB×age: *F*_1,2388_ = 1.24; p = 0.27). Lay date did not influence provisioning rate (lay date: *F*_1, 2913_ = 0.23; *p* = 0.63) nor was there an interaction between lay date and EB (lay date×EB: *F*_1,2387_ = 0.21; p = 0.65). Provisioning rates showed a quadratic relationship with time of day (hour^2^: *F*_1, 2919_ = 928.00; *p*<0.0001), with frequency peaking at dawn and dusk.

**Figure 1:**
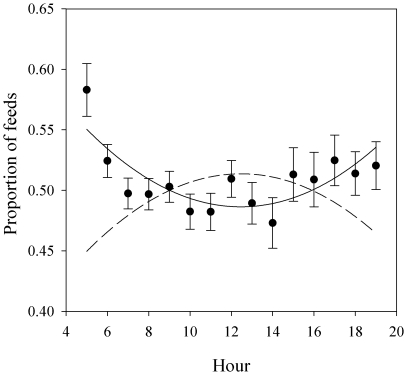
The quadratic relationship between the proportion of visits by a female shown by solid line predicted from the model. Points show the raw mean and standard deviation of all females in the population. The dashed line shows the proportion of feeds by males. The first and last hour are excluded as these are incomplete provisioning hours and feeding behaviour is confounded by roosting behaviour (N = 85 females; 80 males).

The proportion of total pair feeds contributed by the female was not explained by the EB of either parent (male: *F*_1, 1092_ = 1.64; *p* = 0.20; female: *F*_1, 1135_ = 1.24; *p* = 0.26), nor the interaction between the EB of the pair (male EB×female EB: *F*_1, 917_ = 2.18; *p* = 0.14). The proportion of total pair feeds contributed by the female was not explained by female age (female age: *F*_1, 1359_ = 0.83; *p* = 0.36), male age (male age: *F*_1, 1359_ = 0.30; *p* = 0.59) nor the interaction between male and female age (male×female age: *F*_1, 1357_ = 3.60; *p* = 0.06). Furthermore there was no interaction between female EB and female age (female EB×female age: *F*_1, 1135_ = 2.31; *p* = 0.13) nor male EB and male age (male EB×male age: *F*_1, 1092_ = 3.12; *p* = 0.08). However, the proportion of pair feeds given by the female changed in a U-shaped fashion over time, such that females provided more at the beginning and end of the day, relative to their partner (hour^2^: *F*_1, 1359_ = 46.16; *p*<0.0001; Figure 1).

### Plasticity in provisioning behaviour

**Figure 2:**
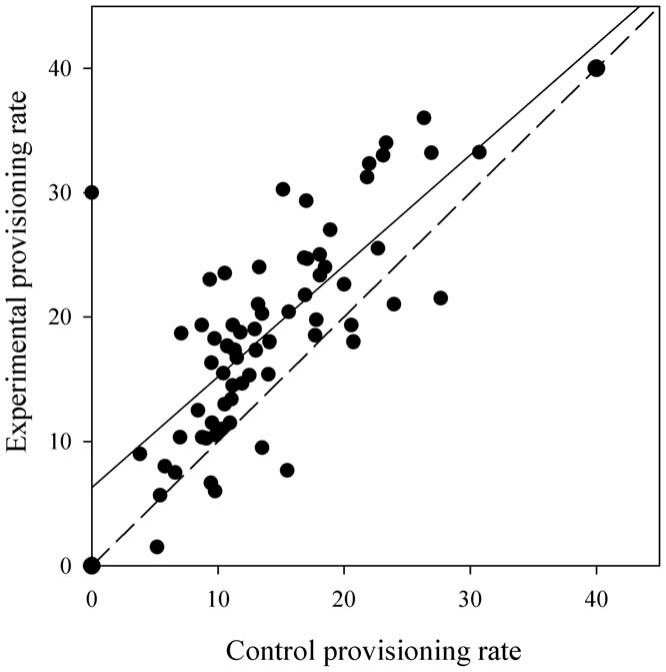
Manipulated individual provisioning rates plotted against control individual provisioning rates. Each circle represents a single individual. The solid line shows the regression slope through the actual data (y = 0.89x+6.18) while the dashed line shows the relationship expected if there were no effect of manipulation. As brood sizes were increased by 33%, a gradient of 1.33 would represent a proportional increase in effort, relative to the control feeding rate. Whereas a gradient of 1 with an intercept greater than zero would show a constant increase in feeding rate in response to the manipulation (N = 65).

Brood demand was manipulated by increasing the brood size by 33% and the subsequent change in provisioning rate measured for the following three to six hours. The first hour post-manipulation was excluded from analysis to reduce potential effects of human disturbance. Parents increased their feeding rate in response to enlarged brood size (treatment (control vs. enlarged brood): *F*_1, 1533_ = 74.79; *p*<0.0001; Figure 2), after controlling for time of day (hour^2^: *F*_1, 1538_ = 89.34; *p*<0.0001). However, the response to greater brood demand was not dependent on a linear relationship with EB (EB×treatment: *F*_1, 1264_ = 2.39; *p* = 0.12) nor a quadratic relationship (EB^2^×treatment *F*_1, 1264_ = 0.05; *p* = 0.83), and there was no significant interaction between EB and sex (EB×sex×treatment: *F*_1, 1244_ = 0.02; *p* = 0.88). There was no interaction between age and response to the manipulation (age×treatment: F_1,1514_ = 0.52; p = 0.47) nor EB, age and treatment (EB×age×treatment: F_1,1245_ = 0.56; p = 0.45). Parents of both sexes responded similarly to increased brood demand (sex×treatment: *F*_1, 1532_ = 0.46; *p* = 0.50). Further analysis found that the increase in feeding rate was not proportional to the increase in brood demand (33%, Figure 2). Instead, individuals with lower unmanipulated provisioning rates showed a significantly greater increase in feeding rate (*t* = 2.89; *p* = 0.005), after taking hour and natural brood size into account .

The change in feeding rate post-manipulation, calculated by dividing the data into 10 minute blocks, was not associated with variation in EB (EB: *F_1_*_, 1276_ = 2.01 *p* = 0.16), nor the interaction between EB and time block (EB×time block: *F*_1, 1277_ = 1.32; *p* = 0.25) nor the interaction between EB and time block^2^ (EB×time block^2^: *F*_1, 1276_ = 1.00; *p* = 0.32). Change in feeding rate was also unaffected by sex (sex: *F*_1, 1488_ = 2.25; *p* = 0.13) and age (age: *F*_1, 1467_ = 0.02; *p* = 0.88). Instead, all individuals initially increased their provisioning rates before reaching a plateau (time block^2^: *F*_1, 1488_ = 6.69; *p* = 0.01), after controlling for the influence of date (Julian day: *F*_1, 1488_ = 5.43; *p* = 0.02).

## Discussion

Despite mounting evidence that personality differences should influence parental care [21], this study found no association between the exploration and provisioning behaviour of great tits. Fast- and slow-exploring individuals fed nestlings at comparable rates and showed similar responses to experimentally increased brood demand. In addition, males and females showed no difference in their average provisioning rates measured over the course of the day, even though the sexes did vary temporally in their relative contributions to feeding young. When brood demand was experimentally increased, both parents were able to provision at a higher rate within a short time period. Interestingly, the magnitude of increase was partly determined by control feeding rate, suggesting that some individuals tend to feed closer to their personal maximum than others.

This is the first study to investigate whether an index of personality correlates specifically with provisioning behaviour in birds, and is one of only a handful of studies of personality and parental care in other wild animals. Individuals in the focal population of great tits have been shown to vary in how quickly, and hence how thoroughly, they explore a novel environment, and that these relative differences remain stable over time and are heritable [25]. Theoretical studies have suggested that consistent differences in behaviour may be favoured by different life-history strategies [27], [28]. Hence, fast-exploring, risk-taking individuals may value current breeding attempts more highly than future ones and so should invest more in caring for current young than their slow-exploring counter-parts. Empirical data from other species are consistent with this idea: bolder convict cichlid (*Cichlasoma nigrofasciatum*) fathers devote more time to parental care [29]; while mice (*Mus musculus*) mothers selected to be more aggressive are more attentive to their pups [30]. Moreover, studies of great tits have found that EB is associated with variation in reproductive success [17], [23], [25], which is likely to be mediated, at least in part, by provisioning of young. Yet contrary to expectations, this study found no evidence for an association between EB and any of the components of provisioning behaviour examined here.

There are several potential explanations for the lack of relationship between EB and provisioning behaviour observed in this population. First, EB may influence aspects of provisioning behaviour not considered in the current study, such as the type or size of food fed to young [31] or the distance flown to find food [32]. For example, parents have been found to vary prey type rather than rate in response to elevated brood demand in starlings (*Sturnis vulgaris*; [31]). Thus, while all great tits increased their feeding rate in response to greater brood demand, slow-exploring individuals may also bring larger prey items since they are better able to exploit new food sources [16], [26]. Future studies may therefore benefit from investigating the provisioning behaviour of parents in greater detail.

Second, although correlations have been identified between EB and behaviours likely to influence parental care, such as foraging, in a Dutch population of great tits [22], [26], [33], these may be specific to an ecological niche that does not occur in the UK population studied here. Research from other taxa suggests that behavioural correlations are not necessarily a property of a species, instead they can vary among populations [34], [35], [36]. For example, the positive relationship between EB and aggression found in three-spined sticklebacks (G*asterosteus aculeatus*) is absent in populations with a low predation risk [35]. This explanation is consistent with the recent finding that the UK population of great tits in the current study experiences a different selective regime to the Dutch population [25], which could either be a cause or a consequence of different behavioural correlations. Thus, EB may not influence parental care in the UK population of great tits simply because EB is not associated with ecologically relevant behaviours in this population. This serves as an important reminder that studies of personality should not assume that behaviours which are correlated in one population, such as exploration and aggression, will necessarily be correlated in another [34], [35], [36]. It should also be recognised that even where EB is correlated with other behavioural traits, parental care may still be associated with some personality traits and not others. Therefore, until we know more about how personality can influence ecologically important behaviours, studies should consider as many behavioural components of personality as is feasible.

A final explanation may be that the relationship between EB and provisioning behaviour is specific to contexts not included in the present study. Indeed, there is growing evidence that the fitness consequences of personality traits fluctuate in space and time [24], [25], [37], [38]. In the focal population of great tits, selection acting on EB was found to be weak, heterogeneous and sex-specific [25]. In particular, in 2006, the year we did the brood size manipulation, there was no evidence for selection on EB, mediated through either fledging success or offspring recruitment [25]. Therefore, it is perhaps unsurprising that slow- and fast-exploring parents failed to differ in their response to increased brood demand during this breeding season. One possibility is that differences between bold and shy birds in their provisioning behaviour are masked when food is abundant. Although we did not test this hypothesis directly, our results do not support this idea. An effect of EB did not emerge with advancing lay date, as might be predicted if there was a decline in food availability over the course of the season [39]. A goal of future studies will be to test explicitly whether the ability to care for young does depend on the interaction between EB and environmental heterogeneity. For example, estimates of variation in food availability among territories, such as oak abundance [25], caterpillar numbers or the mass of chicks fledged during previous seasons [19], [23], could be used to test directly whether the effects of EB on provisioning are only apparent on territories where food is scarce. Alternatively, it may be that fast and slow birds tend to settle on territories of different quality, which best suit their individual foraging styles, thereby reducing any differences in provisioning behaviour.

Experimental manipulation of brood demand showed that parents of both sexes were capable of facultative adjustment of care across a period of hours, although the degree of responsiveness was unrelated to EB. Short-term plasticity in provisioning behaviour is likely to be important in the wild due to fine-scale fluctuations in extrinsic factors such as brood demand, partner effort, predation risk, food abundance, competition and weather. The similarity in responsiveness of both parents, in conjunction with the absence of any sex difference in average contributions to care, suggests that males and females alike are selected to adjust their behaviour to meet the demands of their brood. These results are consistent with previous studies of great tits, which also found no sex difference in natural contributions to parental care ([6], [40], [41] but see [42] for general Paridae discussion).

Interestingly, birds which provisioned most increased their provisioning rates by only half as much as bird that provisioned least when brood demand was experimentally increased. This may be because individuals that naturally feed at a higher rate may already be working at maximum capacity and therefore lack the potential to increase their investment. This idea had already been suggested to explain why, in biparental burying beetles (*Nicrophorus vespilloides*), females tend to provide more care, yet are less responsive to changes in larval begging behaviour [43]. Indeed, an ‘energetic ceiling’ (*sensu* [44]) has previously been demonstrated in great tits, beyond which increasing provisioning effort may not yield sufficient fitness benefits. Female great tits do not increase their provisioning rates nor their daily energy expenditure in response to long-term brood size enlargement, although they do reduce their investment at smaller brood sizes [44]. The authors suggest that females were maximally investing in provisioning at their natural brood size, and that this rate was largely determined by the availability of daylight hours. However, this explanation fails to address why, in the current study, some individuals appear to operate closer to the maximum that others. A recent study of hihi (*Notiomystis cincta*) may shed some light on this issue. Hihi parents, supplemented to be in better condition, fed young at a higher rate than non-supplemented parents [45]. Yet, only non-supplemented birds increased their provisioning rate when given a higher quality brood. Although the authors argued that parents in better condition were showing reproductive restraint, an alternative explanation for this difference is that parents in better condition are working at maximum capacity and so were unable to increase their feeding rate, even when given better quality chicks. Therefore, a future challenge remains to determine whether condition underpins individual variation in great tit provisioning behaviour and whether parents in good condition work closer to their energetic ceiling than those in poor condition.

Although there was no evidence for a sex difference in average provisioning rates, the division of labour between males and females did change over the course of the day. This suggests that studies detecting sex differences in care based on only certain parts of the day should be treated with caution. Female great tits may contribute proportionally more during dawn and dusk since only they roost with nestlings (S. Bouwhuis, Pers. comm.) and so may have exclusive access to information relating to offspring need at these times. In addition, male great tits may be constrained from feeding offspring very early and late in the day, since they typically devote these times to singing [46], [47]. Theory predicts that parents should partially compensate for changes in their partner's work rate [8], [48] but when their knowledge of nestling hunger is poor, they may instead match changes in their partner's effort [49]. While the results from our study are consistent with partial compensation models, they also raise the possibility that the probability and/or direction of matching might vary according to the time of day.

In summary, this study found no evidence that individual differences in EB were associated with differences in provisioning behaviour in great tits. Although these results are surprising given the predictions based on other species and even other populations of great tits, they are consistent with results from the focal population which show that selection acting on EB is weak and highly context-specific. This result emphasises the need for studies of personality to be wary of assuming that behavioural correlations are ubiquitous in a species. A challenge for future work will be to identify the underlying causes of such correlations, and thus predict where and when they will occur.

## Materials and Methods

### Ethical Considerations

All work was carried out within the guidelines of the University of Oxford, and UK standard requirements. All methods were approved by the University of Oxford ethical review board and permission for all work in Wytham woods was obtained from Professor Ben Sheldon. Once caught, birds were kept individually in cloth bags and transported by car to the observation rooms within two hours of capture. Birds were housed under natural light and temperature conditions in individual cages (45 cm×45 cm×68 cm) without visual contact. Birds were fed meal worms, sunflower seeds and water *ad libitum*, supplemented by wax moth larvae to ensure a varied diet. Following assays, birds were returned to the location of capture, where there was abundant food at feeding stations to allow adequate mass gain before dusk. Birds were normally kept in captivity for less than 24 hours. Mean individual mass change from capture until release was +1.0±0.73 g which is within the natural range in the wild [50]. We found no negative impact of personality assays, pit tags or cross fostering on the survival of individuals. Birds were brought into captivity under English Nature licenses 20053006, 20062827 and 20073135 and caught and ringed under BTO license C/5203.

This study was carried out on a wild population of great tits (*Parus major*) in Wytham Woods, Oxfordshire, UK in 2005–2007. Great tits are socially monogamous passerines that nest extensively in artificial boxes and are resident in the UK over winter [51]. During September–March, the EB of wild birds caught from this population was measured in captivity, and their chick provisioning behaviour was then measured in the wild during the breeding season (April–June).

### Measuring personality

**Figure 3:**
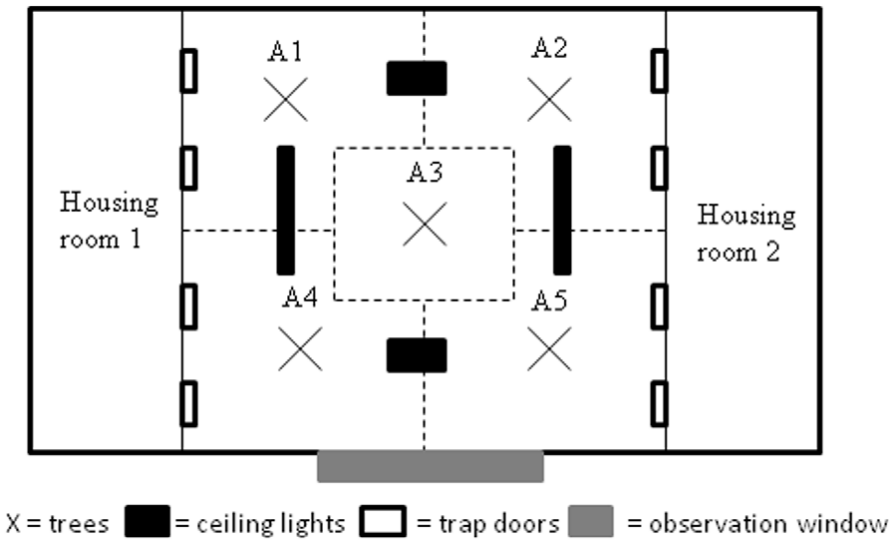
The layout of the experimental room, used to assay personality (see text and [**25**] for detailed description of methodology).

Birds were captured at feeding stations and occasionally off roost at nest boxes. Body mass (±0.1 g) was recorded and unringed birds were banded with a uniquely numbered British Trust for Ornithology (BTO) ring. They were then transferred to the Oxford University Field Station where they were housed individually with food and water *ad libitum*. EB was assayed under standardized conditions in captivity using an adapted open field test, modified from a previously used experimental design [see 16 for original design]. Specifically, birds were released into a novel room, which was split both spatially and by object type (see Figure 3 for layout). Birds were monitored for eight minutes, during which we recorded continuously whether the bird was flying or stationary, and if stationary, which area of the room it was located in and on what type of object (a detailed description of this methodology can be found in Quinn et al. [25]).

### Measuring parental care

From April to June, during 2005–2007, all breeding attempts were monitored throughout a 51 hectare subsection of Wytham Woods [52]. Nest boxes were checked to identify nesting attempts and to record laying date and clutch size. Daily checks were carried out from 13 days after the start of incubation in order to ascertain the exact hatch day (day one). Between day eight and day ten, first year and adult breeders were caught at the nest box using spring-loaded traps, which capture the adult in the box once they enter to feed. Adults were then removed from the box and fitted with a uniquely coded Passive Integrated Transponder (PIT) tag glued to a colour ring. PIT tags enable provisioning rates to be measured remotely (described in detail below) and have been found to have no impact on survival (S. Patrick unpublished data). Once parents had been caught, it was possible to identify which birds were of known personality from ring numbers. Nests where at least one parent had been assayed for EB were subsequently monitored to measure parental care.

Provisioning rates were recorded using an automated system that registers the presence of a PIT tag at the nest box. A circular antenna was fitted around each nest box entrance and connected to a data logger at the base of the nest tree. The unique ID of any PIT-tagged bird passing through the nest entrance was registered by the antenna and recorded on the data logger along with the date and time. While the data loggers simply record the presence of PIT tags, it is possible to derive an accurate measure of provisioning rate using the number of minutes/hour a bird was detected at the nest [53]. Therefore, we used this as a proxy for feeding rate. On day 10, dummy antennae were put up at boxes to allow birds to habituate to the presence of equipment. Data loggers and real antennae were set up at the nest on day 11 between 1100 and 1300 hrs, and provisioning rates recorded for approximately 24 hours. Only one nest abandoned following the appearance of either the dummy or real equipment across three years.

Natural variation in parental provisioning rates were measured in 2005, 2006 and 2007 but in 2006, all broods were also temporarily enlarged following the control observation period described above. Each brood was enlarged by an average of 33%, by adding great tit chicks of the same age from non-experimental nests. Provisioning rates were then recorded for between three and six hours (start time varied from 1100–1500 hours) in the same way as the control provisioning period, after which foster chicks were returned to their original nest. Facultative adjustment in parental care was determined by looking at the effect of enlarging brood size on individual feeding rate. Control provisioning rates of parents did not change between day 11 and 12 (*F*_1, 2917_ = 0.25; *p* = 0.6163), indicating no effect of day. In addition although a change in feeding rate might occur as a response to disturbance at the nest, this seems unlikely because prior to the control observation period, chicks were also removed from the nest for a similar period of time to obtain biometric measures.

### Statistical analyses

All analyses were carried out in SAS 9.1 (SAS Institute Inc, Cary, USA) and non-significant terms removed using step wise elimination. A principal component analysis (PCA) was used to collapse the different measures of EB into a single score, PC1, according to methods described in Quinn et al. [25]. PC1 scores were calculated from behaviour assayed in the laboratory, using a PCA considering the duration of flights, hops, the number of visits to each of five objects and to each of five areas (Table 1). Factor loadings were on average 0.3, which is above the cut off for minimum loadings [54]. Birds became faster explorers as the breeding season approached (F_1, 269_ = 19.15; p<0.0001) and explored more slowly with multiple tests (F_1, 269_ = 6.88; p = 0.0092). For these reasons, EB scores were derived from PC1 scores after correcting for Julian day of test, year and observation number (birds were scored as detailed in [25]). Importantly, the relative differences in EB among individuals persisted, with a repeatability of 0.61±0.05.

**Table 1:** The results from a PCA of EB showing eigenvectors and eigenvalues.

	Prin1	Prin2	Prin3	Prin4
Duration flying	0.3588	0.2777	−0.0298	0.0698
Duration hopping	0.2303	−0.4582	0.3558	0.0132
Area 1	0.3542	0.0539	0.0603	−0.1108
Area 2	0.3650	0.0319	0.0162	−0.0383
Area 3	0.2842	−0.3626	−0.3274	0.2213
Area 4	0.3502	−0.0279	−0.0750	0.1863
Area 5	0.3516	0.1414	0.0708	−0.0113
Object 1	0.1955	0.4327	0.2143	−0.0808
Object 2	0.0520	0.4186	0.2601	0.6303
Object 3	0.3644	−0.2019	−0.3728	0.1024
Object 4	0.2062	0.2251	−0.0648	−0.6896
Object 5	0.1276	−0.3208	0.7029	−0.0871
Eigenvalues	5.4477	1.8422	1.2790	1.0068
Cumulative variation explained	0.4540	0.6075	0.7141	0.7980

The first four principal components are shown as they have an eigenvalue of >1 and cumulatively explain 80% of the variation (N = 1383). The positive loading on all factors for Prin1 shows that the amount of time birds spend flying, hopping and the number of visits to all areas has a positive effect on this score: i.e. it is a measure of exploration.

Data were collected from 94 nests at which both parents were PIT tagged. Of these nests, personality assays were available for 85 females and 80 males, 71 of which were paired. Having carried out an *a priori* power analysis, we determined that a sample of 87 individuals should have been sufficient to detect a relationship between EB and provisioning rate (effect size = 0.15, α = 0.05, power = 0.80). We used two measures of natural variation in care and two measures of plasticity in care as four separate response variables. We selected fixed effects which have previously been shown to influence provisioning rates and used nest and individual identities as random effects to account for the non-independence of repeated measures taken from the same nest box and bird. (1) Individual feeding rate (feeds/hour) was analysed using a general linear mixed model with EB, sex, hour^2^, natural brood size, parental age and year plus all meaningful interactions as fixed effects and individual and nest identity as random effects. (2) Proportion of pair feeds/hour given by the female was analysed using a generalized linear mixed model with a binomial error structure and a logit link function; the number of feeds by the female was set as the numerator and total pair feeds set as the denominator. Female EB, Male EB, hour^2^, natural brood size, parental age and year and all meaningful interactions were included as fixed effects and nest identity as a random effect.

Plasticity in feeding rate following brood size manipulation was investigated as follows: (1) Individual feeding rate (feeds/hour) was analysed using a general linear mixed model with EB, treatment (control vs. enlarged broods), sex, hour^2^, natural brood size, parental age and all meaningful interactions as fixed effects and individual and nest identity as random effects; (2) Rate of change in feeding rate was measured by dividing the nest observation period into 10 minute time blocks following the brood size enlargement and calculating the feeding rate per block. A general linear mixed model was fitted, including EB, sex, parental age and time block^2^ and all meaningful interactions as fixed effects and individual and nest identity as random effects. All means are shown ± one standard deviation, unless otherwise stated.
